# High‐Frequency Spinal Cord Stimulation at 10 kHz for the Treatment of Nonsurgical Refractory Back Pain: Design of a Pragmatic, Multicenter, Randomized Controlled Trial

**DOI:** 10.1111/papr.12945

**Published:** 2020-09-26

**Authors:** Naresh Patel, Aaron Calodney, Leonardo Kapural, Rose Province‐Azalde, Shivanand P. Lad, Julie Pilitsis, Chengyuan Wu, Taissa Cherry, Jeyakumar Subbaroyan, Bradford Gliner, David Caraway

**Affiliations:** ^1^ Mayo Clinic Phoenix Arizona U.S.A.; ^2^ Precision Spine Care Tyler Texas U.S.A.; ^3^ Carolina’s Pain Institute Winston‐Salem North Carolina U.S.A.; ^4^ School of Medicine Wake Forest University Winston‐Salem North Carolina U.S.A.; ^5^ Nevro Corp Redwood City California U.S.A.; ^6^ Duke University Medical Center Durham North Carolina U.S.A.; ^7^ Albany Medical College Albany New York U.S.A..; ^8^ Thomas Jefferson University Philadelphia Pennsylvania U.S.A.; ^9^ Kaiser Permanente Redwood City California U.S.A.

**Keywords:** nonsurgical refractory back pain, medical management, spinal cord stimulation, high‐frequency, 10‐kHz SCS

## Abstract

**Background:**

Spinal cord stimulation (SCS) has been shown to provide pain relief for chronic back and leg pain due to failed back surgery syndrome. But many patients with chronic back pain have not had major back surgery or are not good candidates for surgery, and conventional medical management (CMM) provides limited relief. We have termed this condition nonsurgical refractory back pain (NSRBP). Level 1 evidence does not yet exist showing the therapeutic benefit of SCS for NSRBP.

**Objective:**

To compare 10‐kHz SCS plus CMM (10‐kHz SCS + CMM) to CMM alone for treatment of NSRBP in terms of clinical and cost effectiveness.

**Study Design:**

Multicenter, randomized controlled trial (RCT), with subjects randomized 1:1 to either 10‐kHz SCS + CMM or CMM alone. Optional crossover occurs at 6 months if treatment does not achieve ≥50% pain relief.

**Methods:**

Patients with NSRBP as defined above may be enrolled if they are ineligible for surgery based on surgical consultation. Subjects randomized to 10‐kHz SCS + CMM will receive a permanent implant if sufficient pain relief is achieved in a temporary trial. Both groups will receive CMM per standard of care and will undergo assessments at baseline and at follow‐ups to 12 months. Self‐report outcomes include pain, disability, sleep, mental health, satisfaction, healthcare utilization, and quality of life.

**Results:**

Enrollment was initiated on September 10, 2018. Prespecified independent interim analysis at 40% of the enrollment target indicated the sample size was sufficient to show superiority of treatment at the primary endpoint; therefore, enrollment was stopped at 211.

**Conclusions:**

This large multicenter RCT will provide valuable evidence to guide clinical decisions in NSRBP.

## Introduction

Approximately 13.1% of the U.S. population suffers from chronic low back pain (CLBP)^1^ with the accompanying negative effects on mental health, quality of life, and productivity.^2^ CLBP is a major cause of disability and cost to our healthcare system,^1^ accounting for 3.1% to 4.9% of all emergency room visits,^3^ in addition to the significant cost of lost productivity due to CLBP.^4^

The treatments for CLBP are multimodal and range from topical or oral medication, to physical therapy, to injection therapy and spine surgery.^5^ When CLBP has a clear etiology with a surgical target, then spine surgery is potentially beneficial. If a surgical target is not clear, then the CLBP can be termed nonspecific.^6^ The goal of treatment when the underlying cause of the back pain is not identified or not amenable to treatment is to reduce the symptoms and improve quality of life. In these nonspecific cases, there may be a tendency to overuse opioids or pursue inappropriate surgery.^7^, ^8^

Spinal cord stimulation (SCS) has been utilized for treating chronic pain for multiple decades.^9^ Though efficacy with predominant back pain was typically lower when compared with leg pain,^10^, ^11^, ^12^ newer generation SCS therapies have shown improved results with predominant back pain.^13^, ^14^, ^15^ SCS is considered an effective treatment for failed back surgery syndrome (FBSS) and complex regional pain syndrome of the lower extremities.^13^, ^16^, ^17^, ^18^, ^19^ It is also considered a safe and reversible therapy, with serious complications rarely observed.^20^ Kumar et al.^21^ presented a randomized controlled trial (RCT) with a design similar to that of the present study but in an FBSS population, showing that SCS was superior to CMM in a number of clinical outcomes. The published RCTs studying the efficacy of SCS for CLBP have included mostly patients with FBSS but also some surgery‐naïve patients. The efficacy of SCS for surgery‐naïve patients appears promising based on small prospective series^22^, ^23^, ^24^ and subanalysis of larger studies.^13^, ^25^, ^26^

**Figure 1:**
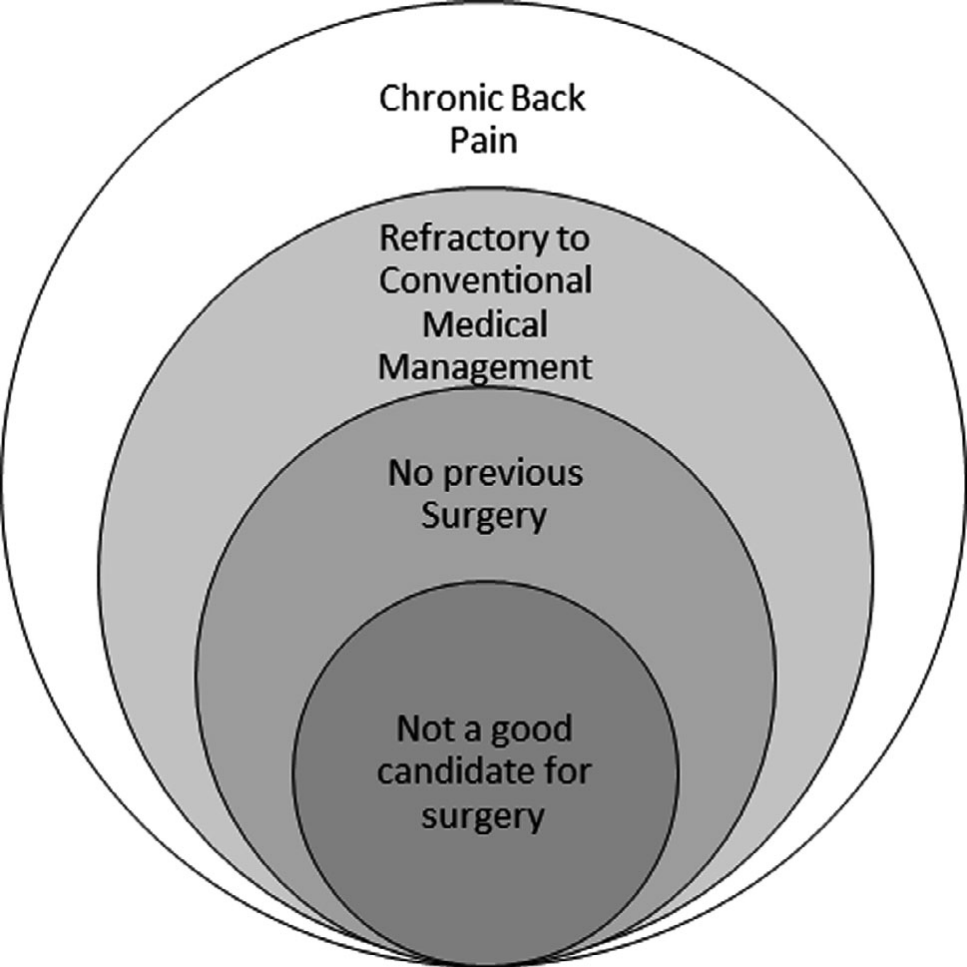
Schematic showing the criteria for non‐surgical refractory back pain population.

A small feasibility study targeting surgery‐naïve patients with chronic refractory back pain who were not candidates for surgery demonstrated significant clinical efficacy out to 36 months in terms of pain, disability, and opioid reduction with high‐frequency SCS at 10 kHz (10‐kHz SCS).^27^ We use here the terminology *nonsurgical refractory back pain* (NSRBP) to represent this patient group (Figure 1). Higher level evidence is needed to demonstrate the clinical efficacy and cost effectiveness of 10‐kHz SCS in this population, which otherwise has limited therapeutic options.^28^

### Study Goals and Objectives

The primary objective of this clinical trial is to evaluate the clinical effectiveness of the addition of 10‐kHz SCS therapy to current conventional medical management (CMM) for NSRBP. Clinical efficacy will be measured in terms of patient‐reported pain relief, disability, quality of life, and change in opioid use. Secondly, the investigation will generate data on the cost effectiveness of the addition of 10‐kHz SCS therapy to CMM in terms of healthcare utilization (HCU) and productivity.

## Methods

The study is a randomized, controlled, multicenter trial investigating the clinical and cost effectiveness of 10‐kHz SCS for NSRBP when used in addition to CMM.^29^ Up to of 216 patients with NSRBP will be randomized 1:1 to receive either CMM alone or 10‐kHz SCS in addition to CMM (10‐kHz SCS + CMM). The total follow‐up period is 12 months for all participants, with an optional crossover at 6 months. Subjects may be withdrawn from the study for reasons such as entry criteria failure, trial failure, subject or investigator request, lost to follow‐up, subject pregnancy, or adverse event. The study protocol and reporting followed Consolidated Standards of Reporting Trials (CONSORT) guidelines, and the outcomes included were based on the guidelines from the Initiative on Methods, Measurement, and Pain Assessment in Clinical Trials (IMMPACT).^30^ The trial registration number is NCT03680846.

### Inclusion Criteria

The study will recruit patients with moderate to severe refractory back pain who have not had previous major lumbar spine surgery, are not candidates for back surgery as assessed by a spine surgeon, or decline surgery. Refractory pain was defined based on a published guideline^31^ as failing to reach treatment goals, which may be related to inadequate pain reduction or daily functioning, or intolerable adverse effects. The complete inclusion criteria are listed in Table 1. The inclusion criteria are designed to be pragmatic to mirror those of nonsurgical patients currently seen in the clinic for whom SCS is indicated. Patients are eligible for the study whether they have unilateral, bilateral, or no concomitant leg pain.

**Table 1:** Inclusion Criteria

Have been diagnosed with chronic, refractory*, * axial low back pain and are not a candidate for surgery based on a spine surgeon’s assessment Pain should have a predominant neuropathic component as per the investigator’s clinical assessment Have not had any surgery for back or leg pain, or any surgery resulting in back or leg pain Considering daily activity and rest, have average back pain intensity of ≥5 out of 10 cm on the VAS at enrollment Be on no or stable pain medications, as determined by the investigator, for at least 28 days prior to enrolling in this study Be 18 years of age or older at the time of enrollment Be willing and capable of giving informed consent Be willing and able to comply with study‐related requirements, procedures, and visits Be capable of subjective evaluation, able to read and understand written questionnaires in the local language, and able to read, understand, and sign the written inform consent

* Pain is defined as refractory, regardless of etiology, when conventional medical management has failed to reach treatment goals that may include adequate pain reduction and/or improvement in daily functioning or have resulted in intolerable adverse effects.

### Exclusion Criteria

The exclusion criteria are designed to mirror the contraindications for SCS implantation in clinical use or pertain to patients being able to accurately report pain for study assessments (Table 2).

**Table 2:** Exclusion Criteria

Have a diagnosed back condition with inflammatory causes of back pain (eg, ankylosing spondylitis or diseases of the viscera) Have a medical condition or pain in other areas, not intended to be treated with SCS, that could interfere with study procedures or accurate pain reporting, and/or confound evaluation of study endpoints, as determined by the investigator Have evidence of an active disruptive psychological or psychiatric disorder identified as the primary condition or other known condition significant enough to impact perception of pain, compliance of intervention, and/or ability to evaluate treatment outcome, as determined by the investigator in consultation with a psychologist Have a current diagnosis of a progressive neurological disease, spinal cord tumor, or severe/critical spinal stenosis Have a current diagnosis of a coagulation disorder, bleeding diathesis, progressive peripheral vascular disease, or uncontrolled diabetes mellitus that would add unacceptable risk to the procedure Be benefitting within 30 days prior to enrollment from an interventional procedure to treat back and/or leg pain*, * Have an opioid addiction or drug‐seeking behavior as determined by the investigator Have an existing drug pump and/or SCS system or another active implantable device such as a pacemaker Have prior experience with neuromodulation devices (SCS, PNS, DRG stimulation, multifidus muscle stimulation) Have a condition currently requiring or likely to require the use of diathermy or MRI that is inconsistent with Senza system guidelines in the physician’s manual Have metastatic malignant disease or active local malignant disease Have a life expectancy of less than 1 year Have an active systemic or local infection Be pregnant (participants of child‐bearing potential that are sexually active must use a reliable form of birth control) Have within 6 months of enrollment a significant untreated addiction to dependency‐producing medications or have been a substance abuser (including alcohol and illicit drugs) Be concomitantly participating in another clinical study Be involved in an injury claim under current litigation Have a pending or approved worker’s compensation claim

CMM, conventional medical management; DRG, dorsal root ganglion; PNS, peripheral nerve stimulation; SCS, spinal cord stimulation.

* Interventions should not be performed less than 30 days prior to enrollment or a follow‐up visit to ensure that the pain level is stable and representative of their long‐term response to CMM.

### Participants

Patients will be recruited in 15 centers in the United States. In all centers, patients who have not obtained satisfactory results with CMM and have not had previous major lumbar surgery will be screened for eligibility by medical record review. Consent will be obtained from potential candidates and the following screening assessments will be performed: back and leg pain on the VAS, magnetic resonance imaging (MRI), x‐rays with or without flexion‐extension views if indicated, and surgical consultation. Inclusion requires a diagnosis of chronic axial low back pain with a neuropathic component. Candidates will complete the PainDETECT questionnaire for assessment of a neuropathic pain component.^32^ The PainDETECT questionnaire will be used to guide the investigators’ assessments, but the score will not be used to automatically exclude potential subjects since the literature is mixed in terms of validating its use in the detection of neuropathic back pain.^33^ Appropriate interventional procedures should have been tried prior to enrollment with continued refractory pain.^31^ All patients were required to have an MRI‐based diagnosis and spine surgical expert opinion that surgical intervention was not indicated. If all eligibility criteria are met, demographics and baseline assessments will be collected, and subjects will be randomized.

### Assignment of Interventions

**Figure 2:**
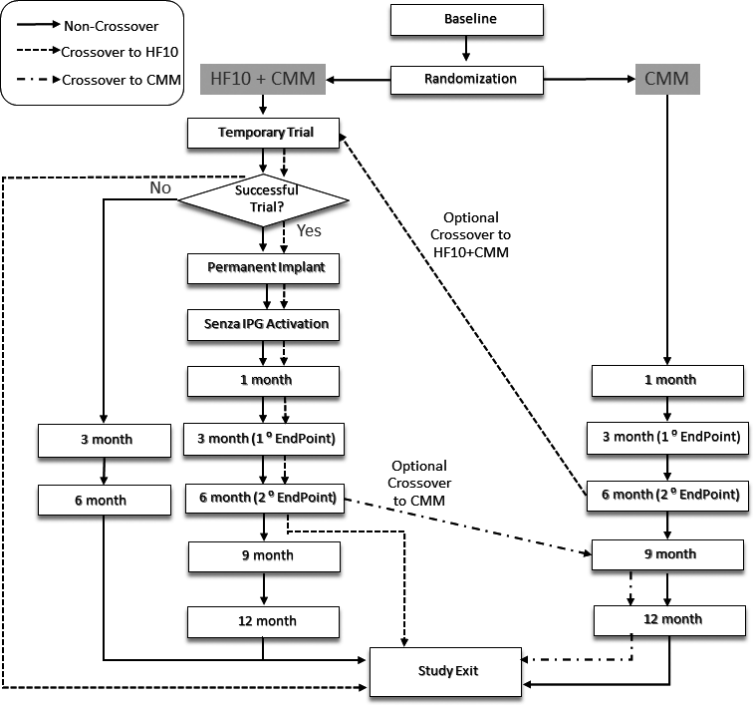
Protocol flow. CMM, conventional medical management; HF10, high‐frequency stimulation at 10 kHz.

Block randomization will be used to help maintain balance in allocation at each site. Randomization assignments will be computer generated and allocated via an electronic data capture system. Randomization is 1:1 to either the 10‐kHz SCS + CMM group or the CMM group. Figure 2 shows the study flow for both randomized groups, and Table S1 shows the timing of assessments.

### 10‐kHz SCS + CMM Group

Subjects randomized to 10‐kHz SCS + CMM will undergo a 14‐day SCS trial phase. Two percutaneous leads with 8 contacts each will be placed in the epidural space spanning vertebral levels T8 to T11.^13^ Stimulation at a frequency of 10 kHz and pulse width of 30 µicroseconds will be delivered from an external pulse generator. The stimulation target and current amplitude will be adjusted until at least 50% self‐reported back pain reduction from baseline is achieved, defined as trial success, or until conclusion of the trial phase. Subjects who pass the trial phase will be scheduled for permanent implantation of the 10‐kHz SCS system (Senza, Nevro Corp., Redwood City, CA, U.S.A.) with investigator and subject agreement. Subjects who fail the trial phase will have leads explanted and will not receive a permanent SCS system but will be followed‐up for 6 months.

The permanent device implantation procedure will be per the manufacturer’s physician implant manual and standard of care. The leads will be placed trans‐fascially and anchored to the fascia, and will be tunneled to a subcutaneous pocket where the implantable pulse generator (IPG) is housed. The IPG is typically implanted in the lower back/buttock region. The device will be activated 0 to 14 days following permanent implantation. The subject will be instructed on the use of the IPG charger and remote control. Programming adjustments will be made at scheduled follow‐ups as needed.

### Conventional Medical Management

All subjects will continue with their CMM, defined as the best standard of care for each individual patient, as determined by the investigator. Table 3 provides examples of therapies that could be part of the investigators’ CMM, but other treatments are not excluded. Previously beneficial treatments may be continued. Conservative care should have been rendered that was generally consistent with the American College of Physicians and the American Pain Society Guidelines as published in the *Annals of Internal Medicine* ^34^ and an interventional pain management guideline from the American Society of Interventional Pain Physicians.^35^

**Table 3:** Conventional Medical Management Exemplary List

Oral medications (including analgesic medication, nonsteroidal anti‐inflammatory drugs, neuromodulating agents, antidepressants) Topical analgesics, compound creams, or counter‐irritants Combined physical and psychological management Physical therapy Back rehabilitation program Spinal manipulation and spinal mobilization Traction Acupuncture/acupressure Cognitive behavioral therapy Nerve blocks Epidural steroid injections Transcutaneous electrical nerve stimulation

### Crossover

Subjects randomized to either treatment group are eligible to cross over to the alternative treatment arm at the 6‐month visit if they meet all the following criteria:

- <50% back pain relief from baseline
- Documented subject dissatisfaction with the treatment
- Investigator agreement with crossover

In the case of a crossover from 10‐kHz SCS + CMM to CMM, the SCS therapy will be switched off and the subject will continue with CMM treatment through the 12‐month visit. If a subject randomized to CMM crosses over to 10‐kHz SCS + CMM, he or she will then undergo a trial and implantation as shown in Figure 2.

### Safety Considerations

The Senza system (including any approved system or component models) is to be used in accordance with its U.S. Food and Drug Administration–approved labeling; therefore, a safety monitoring committee is not required. Adverse events will be assessed at all follow‐ups and any unscheduled visits, and all treatment‐related and serious adverse events will be recorded.

The safety endpoints will be the types and incidence of related adverse events and neurological assessments in each group. All serious adverse events will be reviewed by the principal investigator at each site as they occur and reported in accordance with Medical Device Reporting requirements of 21 CFR 803 when using commercial medical devices.

### Follow‐up

Assessments will be performed at 1, 6, 9, and 12 months for both groups (see Table S1). Unscheduled visits may occur at any time during the study for the assessment of possible adverse events, medication changes, and programming adjustments.

### Data Management and Statistical Analysis

Descriptive statistics will be used to summarize all subject baseline and outcome data collected during the study. Continuous variables will be summarized using means, standard deviations, and ranges. Categorical variables will be summarized in frequency distributions.

Analysis populations defined for the study include intention‐to‐treat (ITT) and per protocol (PP). The ITT population includes all randomized subjects, while the PP population includes subjects who completed the visit corresponding to the endpoint being analyzed. Note that subjects who fail the temporary trial will be included in the ITT population for the high‐frequency stimulation at 10 kHz (HF10) + CMM arm.

The primary endpoint will be evaluated with a Fisher’s exact test comparing the percentage of subjects in each group who achieve a 50% improvement in their back pain VAS score at the primary efficacy assessment in the ITT population. The following secondary endpoints will be successively evaluated (hierarchical closed test approach) at study completion in the order shown with a 0.05 significance level for difference between groups in the PP population until statistical significance is not achieved.

The comparison between HF10 + CMM and CMM alone in terms of healthcare resource use (HRU) will be made based on doctor’s office visits, emergency room visits, or hospital stays, as well as on medical tests and procedures. Medication use (opioids and medication for CLBP), work status, health status (12‐Item Short Form Survey [SF‐12]), quality of life (EuroQol 5‐Dimension, 5‐Level Instrument [EQ‐5D‐5L]) will be used in the cost‐effectiveness analysis. The change from baseline to 6‐month follow‐up will be compared for CMM vs. HF10 + CMM for all these parameters. In addition, subjects can be used as their own control comparing HRU at baseline to HRU after baseline for the HF10 + CMM arm, or pre‐ and post‐crossover for those crossing over from the CMM to HF10 + CMM at 6 months. Parametric functions (eg, Weibull, Gompertz, etc.) will be used to extrapolate from 6‐month HRU to 1, 2, and 3 years out. HRU will be associated with direct medical costs using fee schedules, Medicare claims, and peer‐reviewed literature. Changes in EQ‐5D‐5L index scores will be used to assess changes in health status and the economic value of the addition of HF10 to CMM.

Observational outcomes will be compared between groups using statistical tests appropriate to the type of outcome.

Additional exploratory analyses may be performed to examine the consistency of results in selected subgroups (eg, based on gender, study site, age, body mass index, mental health [based on Patient Health Questionnaire (PHQ‐9) response], pain duration, pain severity, etc.). These analyses may also take the form of multivariable analyses, where the contributions from membership in multiple subgroups to a study endpoint are simultaneously estimated.

### Sample Size Calculation

Assuming a 60% responder rate in the stimulation group (10‐kHz SCS + CMM) and 36% in the control group (CMM), a sample size of 98 subjects in each group is enough to detect a significant difference with a power of 90% and a 2‐sided type I error of 0.05. Assuming a 10% attrition rate, a total of 108 subjects per group need to be randomized.

The sample size computation is based on the 2‐sided Fisher’s exact test, used for comparing 2 independent proportions, following an equal allocation randomization ratio of 1:1.

Sites will be enrolling in the study a maximum of 54 subjects (25%) per site of the total number of study subjects (216 subjects).

### Missing Data

Since the primary analysis of the primary study endpoint at 3 months will be in the ITT population, a sensitivity analysis will be performed to examine the impact of any missing data on the observed results. This sensitivity analysis will use the multiple imputation method for estimating missing primary endpoint data, based on available baseline, 1‐month, and 3‐month follow‐up data.

All other analyses of secondary, tertiary, and exploratory analyses will be based on the PP population and available data. In the case of questionnaires with established rules for scoring results in the presence of isolated missing items, those rules will be followed.

### Interim Analysis

A single interim analysis is planned for when approximately 40% of the planned study population reach the 3‐month primary endpoint. The objective of the interim analysis will be to assess the primary endpoint in order to potentially stop the study early for efficacy or futility, or to increase the sample size for the second part of the study. The overall alpha of 0.05 will be split so that the alpha required to reject the null hypothesis at both the interim and final analysis will be 0.025.

The interim analysis will be carried out by an unblinded independent third‐party statistician who will follow the above‐mentioned procedure and make a recommendation without disclosing interim analysis results or the conditional probability of success to the sponsor.

### Quality Assurance

All data will be collected by the investigational site’s research personnel. Paper data collection forms will be used for patient questionnaires and then transcribed in a secure online database with range checks for data values. The data source will also include the patient’s medical records.

Monitoring visits to the clinical sites will be made periodically for the purpose of ensuring that investigators and their staff understand and accept their defined responsibilities, assessing compliance with Current Good Clinical Practices guidelines, evaluating clinical trial progress, assessing the continued acceptability of the clinical site facilities, assessing compliance with this investigational plan, and verifying the data recorded on the electronic case report forms.

### Duration of the Project

The expected duration of the study is 28 months, followed by an estimated 4 months for data monitoring, addressing queries, data lockdown, and analysis. The time commitment for the participants is approximately 14 months, including eligibility visits, an additional month for trials and permanent implants for the 10‐kHz SCS + CMM group, and follow‐up duration of 12 months.

### Project Management

The study recruitment will be done by investigators and site research coordinators, from patients currently being treated at the site, or referred from primary care, spine surgeons, or other spine care specialties. The principal investigators will verify eligibility and be responsible for conducting the study based on the clinical investigation plan and good clinical practice. Investigators will obtain consent, perform the trial and implantation procedures, perform the neural assessments, and rate the severity of adverse events. Research coordinators will obtain consent, collect study data, and administer questionnaires. Nevro field clinical engineers will provide device support and optimize programming as directed by the investigators.

### Ethics

Consent will be obtained from all potential subjects by technicians at the investigating site, and the study will be conducted in accordance with the U.S. Code of Federal Regulations and recommendations guiding physicians in medical research involving human subjects by the World Medical Association Declaration of Helsinki. The study protocol and informed consent forms will be approved by each study site’s institutional review board (IRB) or by the central IRB (Western Investigational Review Board).

## Results

### Trial Status

The first patient was enrolled on the September 10, 2018. Fifteen U.S. sites are actively participating in the study. As specified in the statistical analysis plan, an independent interim analysis was performed after greater than 40% of subjects reached their 3‐month follow‐up visit. Included in the interim analysis were 101 subjects: 43 randomized to HF10 + CMM and 58 randomized to CMM alone. A Fisher’s exact test was performed to compare the proportion of responders (defined as 50% improvement from baseline) between the CMM alone arm and the HF10 + CMM arm. This analysis was performed on the ITT population. The recommendation based on the results of the planned interim analysis was to stop enrollment in the study because it was already adequately powered to achieve the primary objective at the end of the trial. Therefore, enrollment was stopped at 211.

### Expected Outcomes of the Study

The primary endpoint of this study provides a comparison of the percentage of subjects in each group who experience at least 50% reduction in pain intensity (as assessed by the VAS) for back pain at 3 months compared to baseline.

The secondary efficacy endpoints will be evaluated at the 6‐month time point using a hierarchical test procedure, with the hierarchy being the order in which the endpoints are listed in Table 4.

**Table 4:** Secondary Endpoints

The percentage of subjects with at least a 10‐point decrease from baseline in Oswestry Disability Index score between the treatment and control groups at 6 months Percentage change from baseline in back pain intensity (as assessed by the VAS) comparing the treatment and control groups at 6 months Proportion of subjects reporting “better” or “a great deal better” on the Patient Global Impression of Change questionnaire comparing the treatment and control groups at 6 months Health‐related quality of life evaluation as measured by the EuroQol 5‐Dimension, 5‐Level questionnaire comparing the treatment and control groups at 6 months Change from baseline in opioid equivalent medication usage in each group at 6 months*

* Based on self‐report opioid diary for record of daily opioid intake during the 7 days prior to each follow‐up visit.

The multiple observational outcomes that will be assessed (Table 5) provide significant value since they have not been collected previously in this NSRBP population. The instruments that will be used are described in Table 6. In addition to patient‐reported pain intensity, the effect of pain on daily function and sleep are assessed by the Oswestry Disability Index and the Pain and Sleep Questionnaire 3‐item index, respectively. In addition, improvement in function will be quantified with a “50‐foot walk at fastest speed” test. A composite pain and disability endpoint will allow evaluation of the proportion of subjects in each group who achieve clinically significant improvement in both pain and function.^36^

**Table 5:** Observational Outcomes

Category	Outcome*
Composite pain relief and function	Percentage of subjects in each group with ≥50% reduction in back pain plus a 10‐point reduction from baseline in the Oswestry Disability Index (ODI)
Pain	Percentage change from baseline in back pain intensity (as assessed by the VAS) Percentage of subjects in each group who experience a back pain intensity VAS score of ≤3.0 cm for at least 6 months (remission definition)^36^ For subjects who have baseline leg pain VAS scores ≥ 5.0 cm, percentage change from baseline in leg pain intensity For subjects who have baseline leg pain VAS scores ≥ 5.0 cm, percentage of subjects in each group who experience a leg pain intensity VAS score of ≤3.0 cm for at least 6 months (remission definition)^36^ Changes in Short‐Form McGill Pain Questionnaire
Disability	Percentage change from baseline in ODI score comparing the treatment and control groups Proportion of subjects who experience at least a 10‐point reduction in the ODI score Change in proportion of subjects reporting <20 (minimally disabled) on the ODI total score
Quality of life	Health‐related quality of life evaluation comparing treatment and control groups as measured by the EuroQol 5‐Dimension, 5‐Level questionnaire Mental and physical health evaluation (12‐Item Short Form Survey) comparing treatment to control groups Measure of depression severity comparing treatment and control groups as measured by the Patient Health Questionnaire‐9
Global impression of change	Subject’s and clinician’s impressions of change in overall health condition in each group as measured by the Patient Global Impression of Change and Clinician Global Impression of Change questionnaires, respectively
Satisfaction	Assessment of subject's satisfaction with treatment in each group
Performance	Comparison of length of time to complete 50‐foot walk at fastest speed test
Sleep	Changes in sleep as determined by Pain and Sleep Questionnaire 3‐item index in each group
Work	Missed work days by subject or caregiver Change in subject’s work status in each group Percentage of subjects who have returned to work in each group
Medication	Change from baseline in opioid equivalent medication usage in the subpopulation prescribed opioids at baseline in each group^†^ Change in opioid side effects as measured by a Numerical Opioid Side Effect assessment tool in the subpopulation prescribed opioids at baseline in each group
Healthcare utilization	Between group comparison of number of office visits, emergency room visits, hospital admissions, and medical interventions during the months prior to enrollment and during study follow‐up

* The time frame for all will be 3, 6, and 12 months. Due to optional crossover at 6 months, the 12‐monthanalysis will be “within group” only.

^†^ Based on the self‐report opioid diary for record of daily opioid intake during the 7 days prior to each follow‐up visit.

**Table 6:** Outcome Assessments: Clinical Effectiveness

Measure	Description	MCID
Pain VAS^43^	Pain severity rating. Range: 0 (no pain) to 10 (worst pain imaginable)	1.8 to 1.9 cm^36^
Oswestry Disability Index^44^	Measure of pain effect on different aspects of life. Range: 0 (minimal disability) to 100 (severe disability, eg, bedridden)	10 points^36^
Opioid daily dose	Opioid medication daily dosage. Expressed in morphine milligram equivalents	Opioid daily dose stratifications related to risk have been published^45^
Numerical Opioid Side Effect assessment^46^	Assessment of opioid side effect. Range: 0 (no effect) to 10 (maximal effect)	Not published
McGill Pain Questionnaire: the Short‐Form McGill Pain Questionnaire version 2 (SF‐MPQ‐2)^47^	Intensity of each of 22 pain descriptors. Range: 0 (“do not experience, or none”) to 10 (“worst possible”)	Patients reporting improvement on Patient Global Impression of Change had an average 1.0 point or greater reduction in SF‐MPQ‐2 score compared to baseline than patients who reported no change^48^
Pain and Sleep Questionnaire 3‐item index (PSQ‐3)^49^	Impact of chronic pain on sleep. Each item scored from 0 to 10. Total score range: 0 to 30 points	5.6‐point reduction in PSQ‐3 for back pain patients following treatment^49^
Global Impression of Change by clinician and patient	7‐point Likert scale rating change in activity, limitations, symptoms, emotions, and overall quality of life. Range: “no change” to “a great deal better”	Has been used to determine clinically important difference if patient’s rate “much improved” or “very much improved”^50^, ^51^
EuroQol 5‐Dimension, 5‐Level Instrument (EQ‐5D‐5L)^52^, ^53^	Health state in 5 dimensions is converted to an index value. Range: 0.000 (death) to 1.000 (perfect health); negative values are possible (worse than death)	0.08^54^
12‐Item Short Form Health Survey (SF‐12)^55^	Physical composite score (PCS) and mental health composite score (MCS) are computed using the scores of 12 questions and range from 0 to 100, where 0 indicates the lowest level of health and 100 indicates the highest level of health	8.1 points for SF‐12 PCS, 4.7 points for SF‐12 MCS^56^
Patient Health Questionnaire‐9^57^	Assessment of depression with chronic pain. Scores each of 9 criteria from 0 (not at all) to 3 (nearly every day). The maximum possible score = 27. Scores of greater than 5, 10, 15, and 20 represent, mild, moderate, moderately severe, and severe, respectively.	50% decline and a score less than 10^58^
Physical performance measure: the 50‐foot walk at fastest speed^59^, ^60^	Speed to walk 50 feet when instructed to walk at fastest safe speed	Minimum detectable change is 3.08 seconds based on the coefficient of variation^61^

MCID, minimum clinically important difference.

Quality of life measures (EQ‐5D‐5L, SF‐12), the depression module of the PHQ‐9, and patient satisfaction self‐reported outcome will provide a measure of how the therapy affects overall quality of life and mental health. Mental health is an important outcome because those suffering from chronic low back pain are 3 to 4 times more likely to suffer from depression than the general population.^37^

Opioids are widely prescribed for chronic low back pain, but there is little evidence for their efficacy in the long term.^8^ Prevalences of substance abuse disorders are as high as 43%, and prevalences of aberrant medication‐taking behaviors are as high as 24% in patients with CLBP who are prescribed opioids.^8^ Change in daily opioid use in terms of morphine equivalent dose will be assessed with a patient diary. These data will address whether 10‐kHz SCS can provide an alternative therapy to opioids, avoiding side effects and opioid addiction.

A tertiary objective is to assess the impact of the addition of 10‐kHz SCS therapy on HCU by the patient with NSRBP. This will be the first study to report a cost‐effectiveness outcome comparing 10‐kHz SCS + CMM to CMM in this patient group.

## Discussion

### Theory

The primary endpoint is pain intensity at 3 months using the gold standard of patient report on a VAS,^30^ which is required for labeling claims.^38^ The response definition of at least 50% pain relief permits comparison with other studies and demonstrates separation from placebo, which has been estimated at approximately 30%.^30^, ^39^ The secondary endpoints are at 6 months and include measures of function that are recommended for chronic pain trials.^30^

The safety analysis for this study will include study‐related adverse events, as well as explantations for any cause. Explantations are important to report because they represent additional surgical procedures the patients must undergo, and they represent additional costs to the therapeutic system. Explantation rates have been reported by Stauss et al.^40^ and Van Buyten,^25^ both retrospective analyses, with explantation rates due to loss of efficacy ranging from 1.2% to 5.0%. Although not identified as explantations for the purpose of their analysis, in the Van Buyten article, the rate of explantation due to battery depletion in the nonrechargeable cohort was reported as 37.4% (173/462) within the mean observation period of 2.24 years.

The clinical effect measurement is strengthened by including multiple sites and investigators who are both spine surgeons and pain management specialists, providing a real‐world dataset. The requirement of a surgery consult provides verification of the subject’s surgical candidacy and record of pain etiology.

A study design was published by Al‐Kaisy et al.^41^ that investigated the use of 10‐kHz SCS compared to sham in a more narrowly defined NSRBP population. Inclusion criteria included predominant back pain, degenerative disc disease, and neuropathic pain indicated by the PainDETECT questionnaire. Al‐Kaisy et al. designed the study to compare the incremental clinical efficacy of the therapy over any benefit provided by sham treatment. The study presented here has a different objective: comparing the addition of 10‐kHz SCS therapy to CMM, as defined by the investigators’ standard of care, in a large pragmatically defined NSRBP population. Current clinical referral processes and algorithms for treatment of NSRBP support such an approach.^6^, ^7^, ^8^, ^9^, ^28^ An independent research organization was recruited to interview clinicians in both spine surgery and pain management specialties to generate a definition of NSRBP that is used as the first inclusion criteria. The definition does not call out specific etiologies but defines *nonsurgical* based on the lack of a clearly identifiable surgical target and includes those who are ineligible due to comorbidities or lack of desire to undergo surgery. The pain etiology and reason for ineligibility will be documented so that post‐hoc subgroup analysis of baseline factors that lead to best outcomes may be performed. The pragmatic study population makes the evidence relevant for real‐world clinical decisions and for evaluating true cost effectiveness. We assert that the larger sample size, wider selection of centers (15 vs. 2), and longer follow‐up period (12 vs. 6 months) will result in this study being complementary to the smaller more controlled ongoing Al‐Kaisy study.^41^ Our control group is treated with the best medical management across 15 centers enrolling in this study, including axial and epidural injections, as well as radiofrequency denervations as they pertain to current standards of care.

### Limitations

Due to the nature of the treatment groups, it is not feasible to blind either the subjects or the clinical site personnel to treatment group assignment. Subjects randomized to 10‐kHz SCS + CMM will receive stimulation delivered through an implanted SCS system. The system requires the use of external components for programming and daily recharging, thus precluding subjects from being blinded to their treatment group.

The lack of blinding provides a potential for bias. In preparing the investigational plan, elements have been put in place to anticipate and minimize potential sources of bias.

- The qualifications of each investigator and his or her ability to appropriately screen and treat subjects and to comply with investigational plan requirements will be reviewed before their participation in the trial.
- The study will be conducted under a common protocol at all study sites.
- The sponsor, investigators, and study participants will remain blinded to aggregated study results, except for recommendations made during a single, preplanned interim analysis.
- In order to minimize potential assessment bias, the subjects will receive standard instructions for completing the questionnaires.

One limitation of this study is the potential for a “nocebo effect,” since one arm of this study entails the continuation of the CMM treatment the patients have been receiving. But this effect is mitigated due to the regular care and assessment that the CMM arm group receives within the study. It is likely that some patients will achieve improved treatment results compared to previous CMM in this more intensive care scenario.

In addition, this pragmatic open‐label study is not designed to remove the effect of a subject’s expectations for the therapy or placebo effect. In the practice of real‐world chronic pain treatment, the placebo effect may be considered part of the therapy, “shaping the central nervous system toward pain relief,” since psychological factors are known to influence chronic pain management.^42^

## Conclusion

CLBP disables a significant portion of U.S. adults, negatively impacting their quality of life. Patients with CLBP refractory to CMM who are not surgical candidates (or who decline surgery) lack therapeutic options. Clinicians need evidence to guide recommendations for alternative therapies such as neuromodulation. Given the high cost of health care, it is also important to weigh the economic impact of utilizing 10‐kHz SCS in this group of patients with CLBP who have not had previous back surgery. The aim of this study is to provide high‐quality clinical data that will allow clinicians to make informed choices for their patients with nonsurgical refractory CLBP.

## Funding

This study was funded by Nevro Corp.

## Conflicts of Interest

N.P., C.W., and T.C. have nothing to disclose. J.P. has received a research grant and honorarium from Nevro Corp. and consulting fees from Boston Scientific and Abbott. A.C., L.K., and N.L. serve as scientific consultants to Nevro Corp. D.C., R.P.A., J.S., and B.G. are employees of Nevro Corp.

## Supporting information


**Table S1.** Protocol Assessment Timeline.
