# Supplementary material for: High‐Frequency Spinal Cord Stimulation at 10 kHz for the Treatment of Nonsurgical Refractory Back Pain: Design of a Pragmatic, Multicenter, Randomized Controlled Trial
Source: Pain Pract. 2020 Sep 26;21(2):171–83. doi: 10.1111/papr.12945 (PMC7891432; doi:10.1111/papr.12945)
Supplement: Supplementary file 1 — Table S1. Protocol Assessment Timeline. [file PAPR-21-171-s001.docx]

Supplemental Digital Content. 1. Table. Protocol Assessment Timeline.

| **Assessment** | **Pre-Randomization** | | | **Post-Randomization** | | | | | | | | | | | | | | |
| --- | --- | --- | --- | --- | --- | --- | --- | --- | --- | --- | --- | --- | --- | --- | --- | --- | --- | --- |
| Visit | Enrollment | Entry Criteria^a^ | Baseline Assessment | Trial Implant^b^ | End of Trial  (EoT) ^b^ | Permanent Implant^b^ | Device Activation^b^ (DA) | Telephone Call | 1 Month Visit | Telephone Call | 3 Month Visit | Telephone Call | 6 Month Visit | Telephone Call | 9 Month Visit | Telephone Call | 12 Month Visit | Study Completion |
| Window | - |  | >7 days from Enroll^l^ | - | 0-14 d from Trial Implant | 0-60 d from EoT | 0-14 d from Perm Implant |  | 4 wks ± 7 d from DA |  | 12 wks ± 14 d from DA |  | 24 wks ± 30 d from DA |  | 36 wks ± 30 d from DA |  | 52 wks ± 30 d from DA | 24 or 52 wks ± 30 d from DA^k^ |
| Informed Consent | X |  |  |  |  |  |  | 2-3 Weeks before 1 Month Visit |  | 2-3 Weeks before 3 Month Visit |  | 2-3 Weeks before 6 Month Visit |  | 2-3 Weeks before 9 Month Visit |  | 2-3 Weeks before 12 Month Visit |  |  |
| Medication Usage |  | X^c^ | X | X | X | X | X |  | X |  | X |  | X |  | X |  | X |  |
| Pain Assessment (VAS) |  | X | X^d^ |  | X |  |  |  | X |  | X |  | X |  | X |  | X |  |
| painDETECT |  | X |  |  |  |  |  |  |  |  |  |  |  |  |  |  |  |  |
| Medical/Surgical History |  |  | X |  |  |  |  |  |  |  |  |  |  |  |  |  |  |  |
| Pregnancy Test^e^ |  | [X] |  | [X] |  |  |  |  |  |  |  |  |  |  |  |  |  |  |
| Psychological Evaluation |  | X^f^ |  |  |  |  |  |  |  |  |  |  |  |  |  |  |  |  |
| MRI |  | X^g^ |  |  |  |  |  |  |  |  |  |  |  |  |  |  |  |  |
| Flexion/Extension^h^ |  | [X] |  |  |  |  |  |  |  |  |  |  |  |  |  |  |  |  |
| AP/Lateral X-Rays |  |  |  | X | X | X |  |  | [X] |  | [X] |  | [X] |  | [X] |  | [X] |  |
| ODI |  |  | X |  |  |  |  |  | X |  | X |  | X |  | X |  | X |  |
| Opioid Medication Diary^i^ |  |  | X |  |  |  |  |  | X |  | X |  | X |  | X |  | X |  |
| NOSE^i^ |  |  | [X] |  |  |  |  |  | [X] |  | [X] |  | [X] |  | [X] |  | [X] |  |
| SF-MPQ-2 |  |  | X |  |  |  |  |  |  |  | X |  | X |  |  |  | X |  |
| PSQ-3 |  |  | X |  |  |  |  |  |  |  | X |  | X |  |  |  | X |  |
| PGIC |  |  |  |  |  |  |  |  |  |  | X |  | X |  |  |  | X |  |
| CGIC |  |  |  |  |  |  |  |  |  |  | X |  | X |  |  |  | X |  |
| EQ-5D-5L |  |  | X |  |  |  |  |  | X |  | X |  | X |  | X |  | X |  |
| SF-12 |  |  | X |  |  |  |  |  |  |  | X |  | X |  |  |  | X |  |
| PHQ-9 |  |  | X |  |  |  |  |  |  |  | X |  | X |  |  |  | X |  |
| Subject Satisfaction |  |  |  |  |  |  |  |  |  |  | X |  | X |  |  |  | X |  |
| Neurological Assessment |  |  | X |  |  |  |  |  |  |  | X |  | X |  |  |  | X |  |
| 50 ft. Fast Walk Test |  |  | X |  |  |  |  |  |  |  | X |  | X |  |  |  | X |  |
| Work Status |  |  | X |  |  |  |  |  |  |  | X |  | X |  |  |  | X |  |
| Healthcare Utilization |  |  | X |  |  |  |  |  | X |  | X |  | X |  | X |  | X |  |
| Healthcare Diary |  |  |  |  |  |  |  |  | [X] |  | [X] |  | [X] |  | [X] |  | [X] |  |
| Third-Party Payer Data |  |  | X |  |  |  |  |  |  |  |  |  |  |  |  |  |  |  |
| Adverse Event Monitoring |  | X | X | X | X | X | X |  | X |  | X |  | X |  | X |  | X |  |
| Device Programming |  |  |  | X |  |  | X |  | [X] |  | [X] |  | [X] |  | [X] |  | [X] |  |
| Study Completion^j^ |  |  |  | [X] | [X] | [X] | [X] |  | [X] |  | [X] |  | [X] |  | [X] |  | X | X |
